# Unravelling monocyte functions: from the guardians of health to the regulators of disease

**DOI:** 10.1093/discim/kyae014

**Published:** 2024-08-30

**Authors:** Alexander Mildner, Ki-Wook Kim, Simon Yona

**Affiliations:** MediCity Research Laboratory, University of Turku, Turku, Finland; InFLAMES Research Flagship, University of Turku, 20014 Turku, Finland; Department of Pharmacology and Regenerative Medicine, University of Illinois College of Medicine, Chicago, Illinois, USA; Institute of Biomedical and Oral Research, Faculty of Dental Medicine, Hebrew University, Jerusalem, Israel

**Keywords:** monocytes, macrophages, inflammation, cancer, haematopoiesis and metabolic disease

## Abstract

Monocytes are a key component of the innate immune system. They undergo intricate developmental processes within the bone marrow, leading to diverse monocyte subsets in the circulation. In a state of healthy homeostasis, monocytes are continuously released into the bloodstream, destined to repopulate specific tissue-resident macrophage pools where they fulfil tissue-specific functions. However, under pathological conditions monocytes adopt various phenotypes to resolve inflammation and return to a healthy physiological state. This review explores the nuanced developmental pathways and functional roles that monocytes perform, shedding light on their significance in both physiological and pathological contexts.

## Graphical Abstract

**Graphical Abstract:**
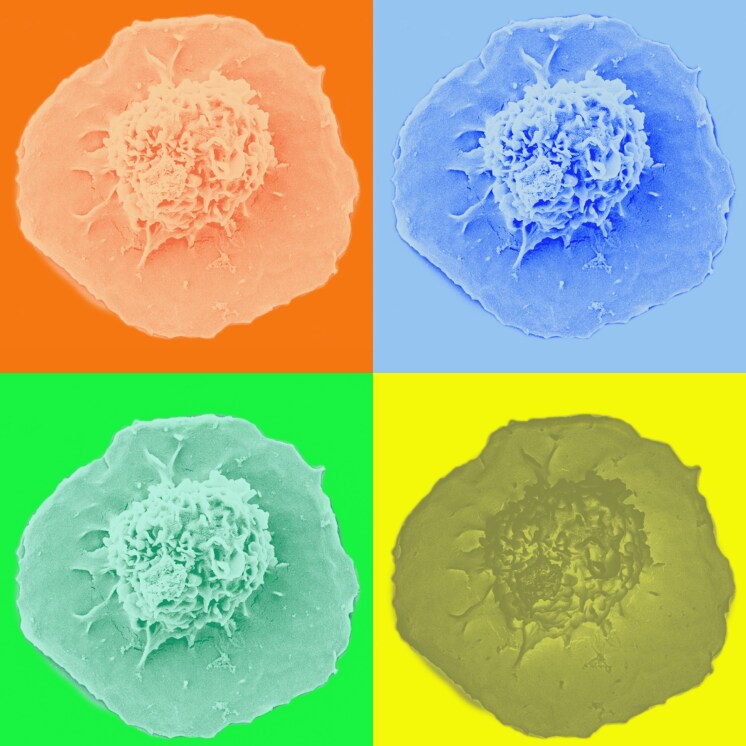

## Introduction

Monocytes constitute one component of the ‘mononuclear phagocyte system’ (MPS) together with macrophages and dendritic cells (DC) [1–3]. Circulating monocytes comprise approximately 10% of peripheral leukocytes in humans and around 4% in mice. It was in 1910 when Artur Pappenheim coined the term ‘the monocyte’ with the goal of consolidating various previously described cell types into a single overarching leukocyte population [4]. Subsequently, the definition of the monocyte evolved to become more stringent, exclusively referring to Ehrlich’s transitional cell [5–9]. Ehrlich described his ‘transitional cell’ featuring a nucleus with a kidney bean-shaped appearance and postulated that these cells are an intermediate state in the developmental process of polymorphonuclear neutrophils. Nevertheless, the introduction of flow cytometry revolutionized immunology and haematology, allowing for the recognition of several monocyte subsets that are conserved across different species [10]. Distinct monocyte subsets were initially identified based on their morphological characteristics and varying expression of CD14 and CD16 in humans [11], a landmark breakthrough in monocyte biology. The combination of CD14 and CD16 membrane expression on human monocytes enabled the classification of three principal human monocyte subsets: CD14^+^CD16^−^ monocytes, also termed classical monocytes, CD14^+^CD16^+^ intermediate, and CD14^Low^CD16^+^ non-classical monocytes. In mice, classical monocytes are defined by the membrane expression Ly6C^Hi^ CCR2^+^ CD62L^+^ CD43^Low^, while non-classical monocytes also described as ‘patrolling monocytes’ are defined as Ly6C^Low^ CCR2^Low^ CD62L^-^ CD43^+^ [12–15] (Table 1). Of note, *Cx3cr1*-driven GFP levels are also often used to characterize and distinguish classical monocytes (*Cx3cr1*^Low^) from their non-classical counterparts (*Cx3cr1*^Hi^) [12], but this distinction reflects the *Cx3cr1* promoter activity and is only suitable for monocyte subset identification in *Cx3cr1*^Gfp/+^ mice. The CX_3_CR1 membrane protein levels on both monocyte subsets appear similar in wild-type mice [16, 17]. The advent of high dimensional cytometry and single-cell RNA-sequencing has further refined the definition of monocyte populations and their origin.

**Table 1:** Phenotype of monocyte subsets

	Cell type	Surface markers (protein level)	Transcription level	Precursor
**Mouse** (CD115^+^, CD11b^+^)	Classical monocytes	Ly6C^high^, CD62L^+^, CCR2^+^, CD177^+^	*Cx3cr1*-Gfp^low^, *Chil3*, *Elane*, *Mpo*	*Ms4a3* ^+^ (GMP)
	Non-classical monocytes	Ly6C^low^, CD62L^–^, CCR2^–^, CD43^+^, CD16.2^+^	*Cx3cr1*-Gfp^high^, *Nr4a1*	Ly6C^high^ monocytes
	Pro-DC3	Ly6C^+^, CD135^+^, CD45RB^+^	*Cd74*, *Cd209a*, *Zbtb46*	*Zbtb46* ^+^ *, Ms4a3^–^* Ly6C^+^ (MDP)
	Cd209a^+^ cells	Ly6C^+^, CD62L^+^, CCR2^+^, CD319^+^	*Cd74, Cd209a*, *Clec10a*	*Zbtb46^–^, Ms4a3^–^* (MDP)
**Human** (HLA-DR^+^)	Classical monocytes	CD88^+^, CD89^+^, CD14^+^, CD16^–^, CCR2^+^	*S100a8/9/12, Vcam*	cMoP (CLEC12A^high^ CD64^high^) within the GMP
	Intermediate monocytes	CD88^+^, CD89^+^, CD14^+^, CD16^+^ HLADR^High^	*Plac8,Hla-dma, Prdm1*	Classical monocytes
	Non-classical monocytes	CD88^+^, CD89^+^, CD14^low^, CD16^+^	*Cdkn1c, Tcf7l2*	Intermediate monocytes
	DC3	CD88^–^, CD89^–^, CD123^–^, FceR1a^+^, CD1c^+^, CD5^–^, CD163^+^, CD14^+/^–^^	*S100a8/9, Vcam, Lyz, Anaxa1*	IRF8^lo^ (CD33^+^ GMP)

Monocytes have long been considered the primary precursor cells for macrophages, capable of replenishing tissue-resident subsets as and when required. Recent mouse studies indicate that monocytes contribute to almost all tissue-resident macrophage pools including the blood (as non-classical monocytes), intestine [18, 19], heart [20, 21], interstitial lung [22–24], serous cavities [25] and possibly even the brain [26]. However, the contribution of monocytes to these pools differs in intensity (from high to low, in the order described) and these conclusions were derived from inbred laboratory mice maintained under specific pathogen-free conditions. Despite potential paradigm shifts in our understanding of the monocyte-to-macrophage development theory—Ralph van Furth *et al.,* recognized in 1972 when defining the mononuclear phagocyte system (MPS) that macrophages could be classified as either free or fixed. They described fixed macrophages as *‘probably of monocyte origin, but definitive proof has not been obtained (...) the morphology and functional behaviour (...) justify their inclusion in the MPS’.* Hence, 50 years ago, it was already acknowledged that certain macrophage populations required continuous monocyte repopulation, while others were potentially more-or-less independent of monocytes [2], a hypothesis that has stood the test of time.

As inflammation and injury ensue, the long-term replacement of embryo-derived macrophages by monocyte counterparts may occur [27]. It remains to be shown how monocytes contribute to tissue-resident macrophage pools in ‘wild’ mammals that regularly experience infections and day-to-day injuries. Understanding how monocytes contribute to the macrophage pool remains challenging to untangle in a human setting. For instance, Langerhans cells in the skin have been shown to remain of donor origin even a decade after a transplant [28]. However, contrasting findings show that following bone marrow transplantation, the majority of Langerhans cells are derived from the donor within just three months [29–31]. It is worth mentioning that the outcomes of these studies could be influenced by environmental, genetic, and/or therapeutic factors, which can create a niche for monocyte-derived cells. Therefore, injury, infection, or ageing in a specific tissue will determine the need to substitute embryonic macrophages with monocyte-derived cells. In addition to their ability to differentiate into tissue-resident macrophages, monocytes serve as an emergency squad during pathological conditions. Accordingly, monocyte accumulation is a common feature of various diseases. Their presence signifies that the homeostatic state has not yet been fully restored.

In this review, we explore the notion that the monocyte has evolved to assist and help, although this may sometimes lead to undesirable consequences.

## Monocyte development and heterogeneity

**Figure 1.**
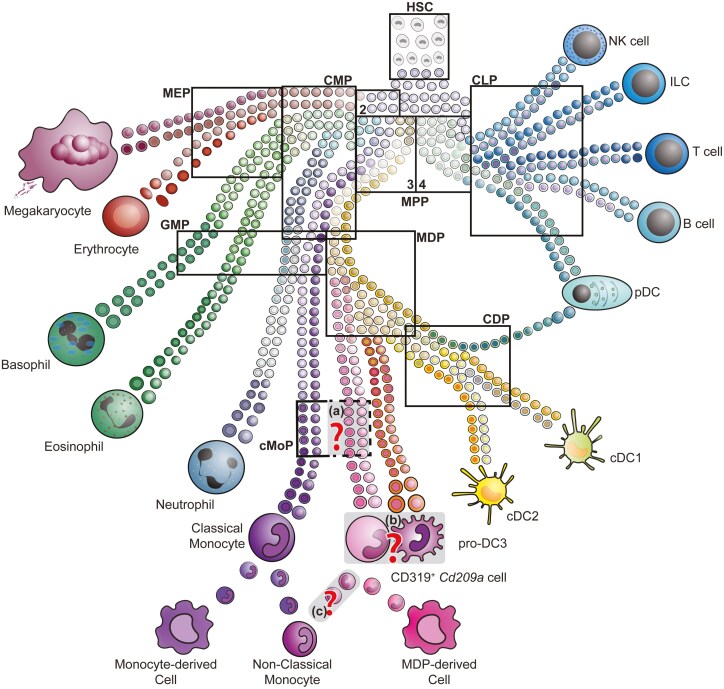
Monocyte development. Haematopoiesis begins with a small number of active haematopoietic stem cells (HSC), which populate a reservoir of multipotent progenitors (MPPs); (indicated by the numbers 2–4 [38]) that already exhibit tendencies toward specific cell lineages. As differentiation continues, precursor populations become progressively more restricted towards specific fates and are classified according to their cellular output and surface characteristics into various precursor subsets (depicted by rectangles), which sometimes exhibit a shared overlapping surface marker profile. Note that the majority of classical monocytes derive from granulocyte-monocyte precursors (GMP), while only a small fraction of Ly6C^+^ expressing cells derive from monocyte and dendritic cell progenitors (MDP). Several open questions remain during monocyte development depicted in grey with question marks: **(a)** Do MDP-derived monocytes go through a common monocyte progenitor (cMoP) stage? **(b)** What is the relationship between pro-DC3 and MDP-derived monocytes? **(c)** GMP- and MDP-derived monocytic cells can experimentally loose Ly6C expression, but do both convert into non-classical monocytes under physiological conditions? Further abbreviations: CLP, common lymphoid progenitor; CMP, common myeloid progenitor; MEP, megakaryocyte-erythroid progenitors; DC, dendritic cell; cDC, conventional DC; pDC, plasmacytoid DC. Figure adapted from [38] with permission.

The process of haematopoiesis was proposed by Alexander Maximow at the turn of the 20th century to be a highly controlled process in which common precursors ultimately generate disparate populations of leukocytes [32]. Ninety years after Maximow’s model of haematopoiesis and three decades after Till and McCulloch delineation of the bone marrow’s ability to generate diverse haematopoietic colonies in the spleen [33], mouse single-cell transplantation experiments using purified haematopoietic stem cells (HSCs) revealed the sustained reconstitution of both myeloid and lymphoid lineages [34]. While these studies showcased the powerful self-renewal capability and multi-potent differentiation potential of HSCs in irradiated mice, the contribution of HSCs to daily haematopoiesis in unperturbed adult bone marrow appears restricted [35–37]. Instead, proliferative short-term HSCs and multi-potent progenitors (MPP) serve as long- and intermediate-term amplifying cells, respectively, and are the main drivers of steady-state haematopoiesis during most of adulthood (Fig. 1). MPPs could be categorized into MPP2-4 oligo-potent states of which MPP3 were mainly a myeloid-biased progenitor [39]. MPP3 can subsequently differentiate into common myeloid progenitors [40] with megakaryocyte/erythrocyte and myeloid potential [41]. The megakaryocyte/erythrocyte differentiation potential is lost in the more restricted granulocyte-monocyte progenitor (GMP) that develops from the CMP (Fig. 1). *Cx3cr1*-expressing monocyte/DC progenitors (MDP) were identified and proposed to be the exclusive precursor of monocytes and conventional dendritic cells (cDC), but not neutrophils [42–44], although conflicting results concerning neutrophil potential within the MDP state were raised [45]. MDPs differentiate into unipotent, proliferative common monocyte progenitors (cMoP) that finally develop into Ly6C^+^ monocytes [46].

However, this prevalent hierarchical view of monocyte and cDC development was recently challenged. Observations by single cell RNA-sequencing (scRNA-Seq) indicated the existence of cells with a gene signature reflecting monocytes or neutrophils already within the GMP and CMP state [47]. Interestingly, the murine MDP population was also identified by flow cytometry within the CMP compartment and may directly arise from an *Flt3*-expressing CMP and not exclusively from a GMP [48]. Seminal studies comparing two distinct inflammatory agents, LPS or CpG [48], indicated that monocytes can arise from two alternative paths: One monocyte differentiation route involves GMPs (also termed ‘neu- or neutro-like monocytes’), while another monocytic subset arises from MDPs (‘DC-like monocytes’), respectively. This was confirmed by clonal barcoding and fate mapping studies highlighting the convergent differentiation trajectories of monocytes [49]. It was also noted under homeostatic conditions that Ly6C-expressing monocytes can be further segregated into MHCII^+^ and MHCII^−^ cells in mice (immgen [14, 50, 51];). The murine MHCII-expressing cells were characterized by a gene profile that corresponds to MDP-derived cells under pathological conditions, e.g. *Cd209a* and MHCII-related genes [50, 51]. The generation of the *Ms4a3*-lineage tracing mouse shed new light on the origin of monocytes. In this mouse line, all cells of GMP origin are labelled when crossed to a reporter line [52]. This system revealed that a large proportion of monocytes (~95% of CD115^+^ cells) are labelled in *Ms4a3*^Cre^  *Rosa26*^*LSL-*tdTomato^ fate mapping mice, while only a small subset (~3–5% of CD115^+^ cells) remain unlabelled [52]. This demonstrates that the majority of monocytes descend from GMPs, which is in contrast to previous studies that clearly showed the potential of MDPs to give rise to Ly6C^+^ cells when adoptively transferred into the blood stream of recipient mice [53]. These data can be reconciled by assuming a dual origin of monocytes in which both GMPs and MDPs have the potential to develop into circulating Ly6C-expressing cells following adoptive transfer. Recent fate mapping and scRNA-Seq experiments further explored the origin of unlabelled Ly6C^+^ cells in *Ms4a3*^Cre^  *Rosa26*^*LSL-*tdTomato^ mice and indicated that these cells correspond to the murine pro-DC3 subset rather than monocytes [23]. The expression of DC-related genes such as *Flt3*, *Zbtb46*, and the superior antigen presentation capabilities support these findings. A CD135^+^ Ly6C^+^ cell population could also be identified in WT mice, which showed antigen-presenting capacity [54]. However, *Flt3* and *Zbtb46* expression could not be detected in another study that identified CD319^+^ MDP-derived cells in the double reporter system *Ms4a3*^Cre^  *Rosa26*^*LSL-*tdTomato^  *Cx3cr1*^Gfp^ [55] (Fig. 1; Table 1), indicating that MDP-derived monocytes and pro-DC3 are two independent cell types. Surprisingly though, the transcriptomic signatures of MDP-derived monocytes by Trzebanski *et al.* [55], show a remarkable overlap with pro-DC3 cells reported in Liu *et al*. [23], while CD177^+^ GMP-derived monocytes correspond to classical monocytes [55] (Table 1). It remains to be resolved if the *Cd209a*^+^ cells [50, 51] are either Ly6C^+^ classical monocytes or pro-DC3 cells [23] (Fig. 1). Curiously, both Ly6C^+^ GMP- and MDP-derived monocyte populations lose Ly6C expression when adoptively transferred into the bloodstream [55], which raises the question if both subsets convert into Ly6C^Low^ monocytes? If so, why do two alternative precursor fates give rise to one homogenous non-classical monocyte compartment [50]? Independent of these artificial transfer experiments, it is important to know, if MDP- or GMP-derived cells exhibit different functions under healthy homeostasis and during pathology. When GMPs and MDPs are injected into the bloodstream of mice with experimental autoimmune encephalomyelitis (EAE), both progenitor cells have the potential to develop into indistinguishable pathogenic subsets in the inflamed central nervous system (CNS) [56], indicating a similar fate when transferred into the same niche (blood) during inflammatory conditions. However, inflammatory stimuli such as LPS or CpG injections favour the generation of GMP- or MDP-derived cells, respectively [48]. Also, the injection of purified GMP- or MDP-derived cells into an empty macrophage niche (in diphtheria toxin-treated *Cx3cr1*^DTR^ bone marrow chimera) gave different results in terms of tissue colonization: while intestinal macrophages were derived equally from either a GMP or MDP progenitor, only GMP descendants efficiently differentiated into meningeal dura mater macrophages [55]. It remains to be determined whether MDP-derived cells belong to the DC family or if they will revert to being classified as a monocyte subset. Future studies will help unify the nomenclature of these populations and hopefully reconcile these conflicting results, as recently attempted in humans. In this setting, DC3 was originally delineated within the human classical monocyte gate, but refinements in flow cytometric analysis helped to identify human DC3 cells and thereby allowed their clear separation from classical monocytes [57–60] (Table 1). However, they still present a challenge for enrichment strategies [61].

Within the bone marrow, monocytes reside near sinusoidal endothelial cells and leptin receptor-expressing perivascular mesenchymal stromal cells. The cells within this niche contribute to the maintenance of monocytes by providing CSF1 [62]. In contrast to the situation in mice, human monocytes rely on FLT3L as patients with a rare *FLT3LG*-deficiency presented a strong reduction of all monocyte subsets in the blood and bone marrow [63]. This phenotype can be attributed to the role of FLT3L in myeloid precursor differentiation and maintenance in the bone marrow [63], but it is unclear if mature human monocytes utilize FLT3L for their survival. Immature bone marrow CXCR4^+^ pre-monocytes undergo further proliferation and differentiate Ly6C^Hi^ CXCR4^-^ monocytes, which up-regulate the chemokine receptor CCR2 [64]. This increased membrane expression of CCR2 is important for the bone marrow egression of classical monocytes through the interaction with CCL2 and CCL7, in mice [65]. However, *CCR2*-deficiency in humans did not affect classical monocyte numbers in the circulation and argues for an alternative egression pathway in humans [24]. In mice, the CCR2-dependent release of monocytes from the bone marrow into the circulation can be influenced by low concentrations of Toll-like receptor (TLR) ligands present in the bloodstream. It was shown that bone marrow mesenchymal stem cells and CXCL12-abundant reticular cells increase CCL2 expression in response to various bacteria-derived TLR ligands, thereby desensitize CXCR4 signalling and inducing the TLR4-dependent monocyte mobilization into the blood [66, 67]. This mechanism secures the rapid mobilization of classical monocytes in the presence of inflammatory signals and demonstrates, how quickly monocyte numbers can adapt to pathological situations. Classical monocytes may also re-enter the bone marrow during circadian oscillation and fasting in a CXCR4-dependent manner [64, 68]. Compared to classical monocytes, non-classical monocytes traffic from the bone marrow into the blood in a CCR2-independent fashion, but they utilize S1PR5 signalling [69].

Taken together, recent studies have revealed that classical monocytes arise from two alternative paths (either through GMP or MDP) under homeostatic and inflammatory conditions. Future research will reconcile these findings and establish a unified understanding of the monocytes that arise from distinct progenitors.

## Monocyte plasticity under homeostatic conditions

The prevailing theory concerning the destiny of classical monocytes upon entering the peripheral circulation is that they possess significant potential for further development before reaching a fully differentiated state [70]. Epigenetic analysis of mouse classical monocytes reveals that this monocyte subset is not characterized by a specific chromatin landscape compared to non-classical monocytes. Only a minor fraction of genes are uniquely accessible and carries histone modifications specific to classical monocytes. In contrast, non-classical monocytes possess a higher degree of unique *de novo* established regulatory elements comprising classical macrophage genes including *Cd36* and *Pparg* [50]. Similar to other tissue-resident macrophage populations, non-classical monocytes are also CSF1 dependent, while the numbers of short-lived classical monocytes are virtually unaffected in mice with a deficient CSF1 signalling pathway [71]. Adoptive transfer of classical murine monocytes into the circulation or following bromodeoxyuridine (BrdU) pulse labelling revealed that classical monocytes quickly lose their phenotypical characteristics and differentiate into non-classical monocytes between 24 and 48 hours [27, 50, 72]. In humans, this developmental profile is conserved, demonstrated through stable isotope labelling. Classical monocytes transition into intermediate and subsequently non-classical monocytes within 4 days [73, 74]. These data suggest that classical monocytes represent a more immature ephemeral monocyte subset that either extravasate into tissues or develops into terminally differentiated non-classical monocytes. The mechanisms involved in how classical convert into non-classical monocytes and how the subsets differ with respect to metabolism and tissue recruitment will be discussed in the following subsections.

### Monocyte conversion

The factors that dictate classical monocyte conversion remain to be fully elucidated. However, recent findings suggest that endothelial niches or micro-injuries could potentially play a role in facilitating classical monocyte conversion into cells adhering to the endothelium and subsequently maturing into non-classical monocytes with reparative abilities. Elegant *in vitro* studies have pointed to an important role in Notch2-DLL1 interaction for this ‘default’ monocyte conversion. When MDP or cMoP were cultured in the presence of *Dll1*-expressing OP9 cells, descendants with classical monocytes and non-classical monocytes characteristics were identified [75]. Nevertheless, GMPs as monocyte precursors were not examined in these studies. It appears that the transcription factor C/EBPα acts as an important negative regulator of the *Notch2*-dependent monocyte conversion program. Deletion of *Cebpa* resulted in a decreased Ly6C^Hi^ monocyte pool and facilitated the direct transition of MDPs into non-classical monocytes. Therefore, absence of *Cebpa* favour’s the alternative generation of monocytes via the MDP lineage [76]. However, it is unclear, if the remaining Ly6C^Hi^ monocytes observed in *Cebpa*-deficient mice are pro-DC3 (or MDP-derived Ly6C^Hi^ monocytes) or if they are ‘escapees’ that can serve as non-classical monocyte progenitors as observed in *Ccr2*-deficient mice. Other factors involved in *Notch2*-dependent monocyte conversion were identified using this *in vitro* system [77] and comprise activity of IRF2 [75]. The necessity of *Notch2* and *Irf2* for monocyte conversion was verified *in vivo* and mainly endothelial cells in the splenic marginal zone and bone marrow were crucial providers of DLL1 [75, 77]. Moreover, TLR7 activation of classical monocytes was also involved in the conversion to non-classical monocytes in a Notch2-independent manner [78]. Other genes that participate in non-classical monocytes generation and maintenance comprise *Nr4a1* [79], *Cebpb* [50, 80], the tyrosine kinase *Lyn* [81], *Cx3cr1-Cx3cl1* [82, 83], and *Bcl6* [75]. Gene deletion of each of these factors leads to the reduction or absence of non-classical monocytes, but the precise genetic interplay between the candidates requires further investigation. The survival of non-classical monocytes depends upon their interaction with the vascular endothelium, mediated through the CX3CL1-CX3CR1 axis, alongside ITGAL (CD11a). This interaction enables the uptake of endothelial-bound CSF1, which is essential for the survival of non-classical monocytes [84]. H-syndrome, a genetic inflammatory disorder that predisposes the patient to histiocytosis, has long puzzled haematologists. This condition is caused by a loss of function mutation in *SLC29A3* that encodes the lysosomal nucleoside transporter ENT3 involved in TLR and MAPK signalling, and results in elevated circulating non-classical monocyte numbers in the blood. Surprisingly, treatment with a MEK inhibitor resolved the histiocytosis, inflammation, and returned the non-classical monocytes back to healthy levels, indicating a crucial role for this lysosomal transporter and MAPK signalling in monocyte homeostasis [85].

### Monocyte fluctuation

Transcriptomic and epigenetic analysis of human monocyte subsets showed that classical monocytes express genes involved in the glycolytic and the pentose phosphate pathway, while non-classical monocytes express genes associated with oxidative phosphorylation [86]. Accordingly, constructing and maintaining large quantities of classical monocytes on a daily basis is energy intensive and requires a tight balance to ensure cost-effectiveness to the organism. Indeed, classical monocytes adapt and respond to external disturbances that influence the energy homeostasis of the organism [87]. Quantitative changes in classical monocyte cell numbers could be observed during high-fat diet consumption [65, 88], circadian oscillation [89], ambient temperature [90], and short-term fasting [91], to name a few. Detailed analyses involved in classical monocyte disappearance during fasting indicated at least two possible molecular mechanisms. First, monocyte egression from the bone marrow is inhibited due to reduced systemic CCL2 levels [91]. Second, classical monocytes are quickly drawn back into the bone marrow via the hypothalamic–pituitary–adrenal axis that regulates CXCR4 expression levels on the classical monocyte [68]. These ‘veteran’ cells persist in the bone marrow and are only released once the energy state is restored. These processes increase the lifespan of classical monocytes and potentially conserve energy that would otherwise be required for the *de novo* generation of a new classical monocyte pool. However, it remains uncertain whether the retracted classical monocytes hinder the generation of new monocytes or if they occupy and obstruct the same cellular niches as newly generated classical monocytes. It is also unclear, how non-classical monocytes respond to these external stimuli, but since non-classical monocytes can adapt their lifespan and accordingly do not rely on a stable and continuous supply of classical monocytes input to maintain their cellular pool [27], it can be speculated that short term classical monocyte fluctuations do not affect more long-lived non-classical monocytes numbers.

### Monocyte recruitment

The migratory ability of classical monocytes, coupled with their adaptable chromatin profile, likely grants them considerable flexibility to respond to external factors and adopt diverse phenotypes. This could explain the intricate differentiation potential observed in classical monocytes compared to non-classical monocytes, which remain within their endothelial niche. As such, classical monocytes develop into tissue-resident macrophages in the intestine [72, 92], dermis [93, 94], heart [20, 21], interstitial lung [22–24], and peritoneal/pleural cavities [25]. In addition, during the colonization of a ‘macrophage-free niche’, e.g. after irradiation, diphtheria toxin-mediated depletion of resident macrophages, infection, injury or even ageing, tissue-infiltrated classical monocytes can acquire tissue-resident macrophage phenotypes dictated by the respective tissue microenvironment [95, 96]. This adaptation under homeostatic conditions is almost complete and monocyte-derived macrophages in the tissue are phenotypically indistinguishable from their embryonic counterparts [95, 97]. However, certain transcriptomic and epigenetic differences may persist in monocyte-derived macrophages, which might stem from their distinct cellular origins. Classical monocyte-derived cells originate from adult monocytes, whereas foetal monocytes and EMP (erythro-myeloid progenitors) give rise to embryo-derived macrophages. Although a detailed comparison of the epigenetic landscapes of adult and embryonic monocytic precursors is yet to be conducted, significant transcriptomic differences have been observed between adult and foetal monocytes [98], which might in turn influence the inflammatory response of classical monocyte-derived cells [99, 100]. It is evident that certain genes appear to be exclusively accessible in embryonic-derived macrophages, e.g. *Sall1* in microglia. In contrast, the chromatin structure of these specific genes remains inaccessible to their monocyte-derived counterparts [100–102]. Surprisingly, monocyte-derived microglia that also expressed *Sall1* and were virtually indistinguishable from embryo-derived microglia were recently identified in aged *Ms4a3*^Cre^  *Rosa26*^LSL*-*tdTomato^  *Cx3cr1*^Gfp^ mice [26], indicating that a complete adaptation of monocytes to the unperturbed niche may be possible.

Another explanation for the differences observed between classical monocyte-derived and embryo-derived macrophages lies within the experimental setting itself, which requires the manipulation and removal of macrophages from their niche to allow monocyte colonization. The methods used to create an available empty niche can have severe side effects, for instance, irradiation-induced DNA damage or substantial cell death in the case of diphtheria toxin-based ablation regimes, which might inadvertently influence the transcriptome of newly recruited monocytes [103].

Building upon the concept that non-classical monocytes may be viewed as terminally differentiated macrophages residing in the blood [27], these monocytes exhibit a reduced tendency to migrate into tissues. Accordingly, non-classical monocytes have only been proposed to differentiate into lung-resident macrophages after adoptive transfer [104]. Additionally, the absence of specialized thymic macrophages in *Nr4a1*-deficient mice correlates with the decrease in non-classical monocyte numbers [105]. However, to confirm if thymic macrophages are bona-fide non-classical monocyte-derived macrophages will require a specific model that targets non-classical monocytes. Instead, non-classical monocytes predominantly remain within the blood, where they are continuously surveying the vasculature by crawling on endothelial cells, which relies on LFA-1/ICAM1 interaction. This process helps to maintain lumenal vessel integrity under physiological conditions [106, 107], while during pathology non-classical monocytes participate in the recruitment of neutrophils [106]. Moreover, the functional difference between monocyte subsets also extends to phagocytosis, where non-classical monocytes exhibit an acidic pH within their phagosome, while classical monocytes maintain an alkaline environment [108, 109]. This suggests distinct differences in the mechanisms by which these two subsets process engulfed material. Further investigations into the enzymes released within each phagosome will provide insight into the specific roles these populations play when encountering pathogens.

In summary, classical monocytes show characteristics of an emergency squad that is able to migrate to any tissue and, when a niche becomes available, differentiate under the influence of local tissue-specific factors to tissue-resident macrophages.

## Monocyte activation and states

**Figure 2.**
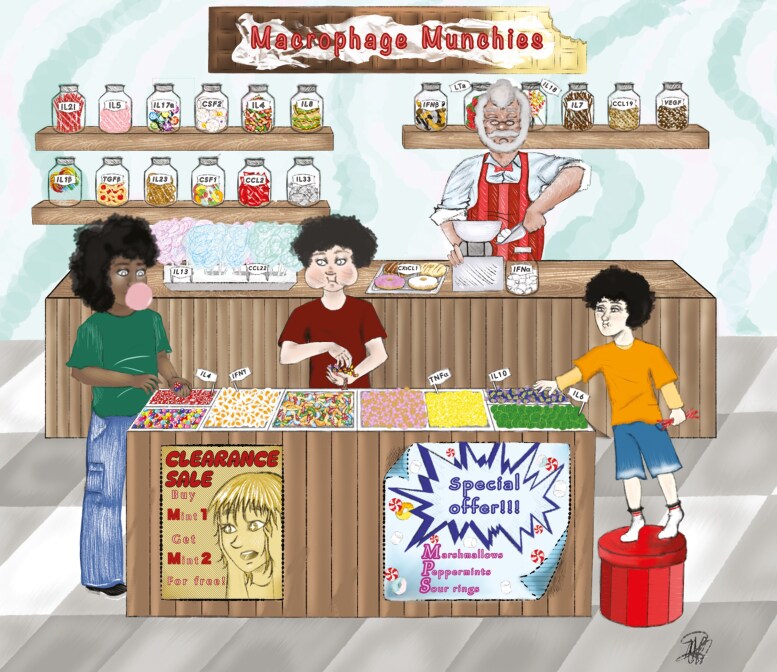
The sweet shop analogy. A sweet shop in a village (organism) represents an organ or an inflammatory environment (infection, injury, or tumour). Frequent visitors to the shop are children (monocytes) who have an extraordinary ability to consume many of the sweets, representing cytokines and secreted mediators that are available in the shop. Each shop offers a different selection of sweets, from lollipops and liquorice to marshmallows, where the range of sweets mirrors the cytokines and chemokines found in different inflammatory microenvironments. For instance, liquorice represents pro-inflammatory TNFα, lemon sherbet stands for IFNγ, while caramel symbolizes anti-inflammatory IL-4. Yet, there are many other sweets on offer, which can simultaneously be tasted by the children, as long as the shop is open (an indication of ongoing inflammation). Under these circumstances, it is unlikely that the children will only eat caramel (IL-4) or lemon sherbet (IFNγ). They will randomly taste other sweets that are available, depending on the architecture of the sweet shop and the sweets on offer. Accordingly, it is possible that the children eat sour lemon sherbet (IFNγ), sweet caramel (IL-4), and bitter liquorice (TNFα) in immediate succession, which all influence the mood and behaviour of the children, reflecting gene expression changes and functional adaptation of the monocyte-derived cells. It is also possible that one child does not like the taste of a particular sweet (such as bitter liquorice), symbolizing the absence or down-regulation of certain cyto- or chemokine receptors on monocytes. Therefore, the *in vitro* single stimulus polarization model may be suitable to precisely control the functional outcome of a single mediator. However, it fails to mimic the complex 3D structured *in vivo* dynamics, the many secreted factors available in a microenvironment, temporal aspects of inflammation, and the array of phenotypes monocyte-derived cells can display during different stages of inflammation, injury, or cancer. Drawing by Maya Rachman.

Monocytes and their descendants are often pigeonholed as pro-inflammatory (M1) or anti-inflammatory (M2) in the literature, suggesting that monocytes primarily fulfil one of two functions. Over 30 years ago, the term ‘alternative activation’ was coined, referring to the inherent plastic nature of macrophages when incubated with the Th2 cytokines IL-4 or IL-13 that increases the expression of the mannose receptor. This activation was indeed in contrast to Mackaness’s ‘classical’ microbial activation that results in the production of inflammatory cytokines [110]. Nevertheless, the up-regulation of pro-inflammatory cytokines or nitric oxide synthases (e.g. *Nos2*) compared to steady-state monocytes may not always yield a reliable conclusion with respect to their pro-inflammatory state, and likewise, the expression of genes such as arginase (encoded by *Arg1*) does not always indicate an anti-inflammatory/regulatory function. This oversimplified classification system neglected the nuanced complexity of monocyte biology and overlooks the dynamic changes that occur over time, particularly in the context of pathological processes. For instance, a single monocyte can express both prototypical pro- and anti-inflammatory markers, such as *Arg1* and *Nos2*, while contributing to tissue damage in conditions such as EAE [111, 112]. Furthermore, a detailed analysis of interstitial macrophages demonstrates that macrophages can respond to the prototypical M1 stimulus IFNγ in a wide variety of activation states that greatly surpass the binary M1 and M2 model [113]. To account for these observations, a spectrum of activation states was proposed, incorporating all intermediate stages between the polarized states from M1 to M2. However, *in vivo* myeloid cells are not only exposed to a variety of cytokine concentrations, but they may also receive a cocktail of various ‘M1’ or ‘M2’ cytokines simultaneously, depending on their local microenvironment and time of infiltration. Analogous to a child (monocyte) in a sweet shop (inflammation) who can not only choose between two specific sweets (e.g. only liquorice or only caramel) but may also experience a wide variety of different flavours, including vanilla, aniseed, mint humbugs, lemon sherbet and chocolate (all synonyms for different cytokines such as IL-10, IL-6, and TNFα; Fig. 2). As long as an active infection or inflammation is ongoing, monocytes can encounter cyto- and chemokines in close proximity that influence their cellular function and phenotype. Under these circumstances, it is very unlikely that monocytes will only sense IFNγ or IL-4. Rather, monocytes will be exposed and sample other mediators present in a random sequential order depending on the location and time of infiltration. Accordingly, it is possible that monocytes react to TNFα molecules, followed by IL-4, and IFNγ in *immediate succession* (Fig. 2). Therefore, the M1/M2 model may be suitable for *in vitro* experiments, where precise control and supplementation of a single mediator is feasible. However, it fails to mimic the complex 3D structured *in vivo* dynamics and temporal aspects. Accordingly, we respectively agree with the viewpoint expressed by our colleagues and kindly advocate for the discontinuation of the M1/M2 model (including any spectrum variants), particularly in the context of *in vivo* macrophage biology [114]. We propose a nuanced exploration and description approach of pathologies, such as sterile, helminth, bacterial or viral infections, aiming to elucidate the intricate dynamics of the unique cytokines within each microenvironment and their profound impact on the activation state of monocytes.

## Monocyte biology during pathology

Monocytes are migratory cells poised to rapidly reach all regions throughout an organism. Functioning as innate immune sentinels, their primary role is to maintain the organism’s physiological health and homeostasis, during infection in terms of inflammatory activity and during wound healing by tissue remodelling. It is also possible that monocytes seed a growing tissue (like a tumour) to fulfil tissue-resident macrophage functions such as apoptotic cell removal or debris clearance. Therefore, the presence of monocytes in a tissue may suggest that homeostatic balance is yet to be completely restored. The presence of monocytes is therefore not per se detrimental to the tissue. Instead, it raises several questions: Why are monocytes present in a tissue under pathological conditions? What factors are different to the homeostatic situation? How do monocytes contribute to restoring the physiological state?

Our way of life has undergone significant changes over the past two centuries. In the Western world, advancements in medicine have led to an increased life expectancy, although with a higher risk of cancer. Physical activity has declined, a disrupted work/life balance contributes to stress and sleep disorders and nutritionally deficient food and beverages are readily available and consumed frequently. The culmination of all these factors influences monocyte biology as mentioned earlier and changes in our lifestyle can contribute or instigate pathological events. For example, in the case of metabolic diseases such as atherosclerosis and obesity, monocytes encounter high concentrations of lipids in arteries or adipose tissue, where a hypoxic environment prevails. As these free or cell-released lipid concentrations exceed the physiological levels, it is possible that monocytes are not equipped with the right molecular machinery or metabolism to perform efficient recycling of the ingested material in the necessary timeframe. The formation of foamy, lipid-laden cells observed under certain metabolic diseases such as atherosclerosis or obesity can point to a restricted catabolic potential that leads to intracellular lipid (mainly cholesterol) accumulation and compromised macrophage activity. The reason for such a phenotype might be the absence of a gene expression program in macrophages that allows efficient lipid meta- and catabolism due to missing niche-derived instructive signals. The endothelial and adipose niches probably did not evolve to produce cytokines that induce a lipid-handling gene signature in macrophages as observed in the lung, where type II epithelial cells produce CSF2 (also known as GM–CSF) to license surfactant catabolic ability to alveolar macrophages [115, 116] since excessive extracellular lipid accumulation is an unnatural phenomena in vessels and adipose tissue.

In other cases, such as autoimmune neuroinflammation (e.g. multiple sclerosis), it is possible that CNS-infiltrated monocytes receive signals that initiate a context-specific neurotoxic differentiation program. This inflammatory phenotype might only manifest in the myelin-rich CNS, while the same educated monocytes execute no tissue-destructive activities in other organs [117]. Therefore, the education and environment are equally important to unleash pathogenic functions of monocytes in specific organs. In addition to the prevalent educational milieu, it has also been reported that different concentrations of the same bacterial stimuli (for instance *Staphylococcus aureus*) can evoke distinctive myeloid responses and functions in monocytes [118], which makes it difficult to a priori predict a uniform, standardized immune response of myeloid cells towards a certain pathogen.

Taking all these factors into account, a detailed analysis of the inflammatory context in terms of prevalent cyto- and chemokines, cellular neighbourhood, immunometabolism, pathogen concentration, and cell origin is necessary to define a statement about the potential functionality of monocytes during pathology. We will discuss some of these aspects in the following sections.

## The role of monocytes during metabolic diseases

### Obesity

Different myeloid cell subsets with distinct functions and origins can be identified in lean and obese adipose tissue. Since this review focuses on monocyte-derived cells under pathological conditions, we refer the reader to two recent, excellent reviews that cover the role of all macrophage subsets during homeostasis and obesity [119, 120]. The first notion that monocyte-derived cells accumulate during obesity in adipose tissue came from bone marrow chimera studies, in which syngeneic CD45.1^+^ bone marrow cells were transferred into irradiated CD45.2^+^ recipients [121]. However, irradiation itself can lead to niche changes and thus influence the results regarding the recruitment of monocytes [122]. When examining the contribution of monocytes to pathology, *Ccr2*-deficient mice have proven an invaluable tool. These mice exhibit reduced circulating classical monocytes, attributed to an egression defect in the bone marrow [123]. Consequently, these mice offer a means to approximate the contribution of classical monocytes to pathogenesis. When *Ccr2*-deficient mice receive a high-fat diet a model of obesity, similar weight gain was observed. However, *Ccr2* deficiency leads to reduced macrophage accumulation in adipose tissue, an attenuated inflammatory milieu, and thereby improved systemic glucose homeostasis and insulin sensitivity [124]. Similar results were observed upon macrophage depletion by clodronate liposomes or in chimeric CD11c^DTR^ mice, which results in decreased accumulation of monocyte-derived cells in adipose tissue [125–127]. Accordingly, the adipose tissue changes reported during obesity were linked to hyperthropy-related hypoxia and the presence of an enriched inflammatory environment [128, 129]. Inflammation indeed contributes to obesity could also be observed in mice with a myeloid-restricted deletion of *Myd88*. These mice showed decreased macrophage recruitment into adipose tissue and were protected from insulin resistance [130]. All these studies led to the hypothesis that the accumulated macrophages are classically activated and that inflammation contributes to tissue damage. The subsequently used term ‘metainflammation’ suggests that pathogen-induced inflammation can be compared to obesity-dependent inflammation in adipose tissue. Recent research, however, shows that this is not the case. Seminal findings demonstrate that the metabolic activation of macrophages results in a unique macrophage phenotype, which is mechanistically distinct from classical pathogen-induced activation [131]. This indicates that metabolic disease-specific pathways influence macrophage activation likely through discrete mechanisms that differ from those involved in pathogen-dependent activation.

Furthermore, recent transcriptomic analysis of mononuclear phagocytes in adipose tissue revealed a high degree of heterogeneity and proposed distinct functions of each subset during obesity development [119, 132]. These data show in terms of monocyte fate that adipose tissue-infiltrated monocytes mainly develop through a sequential differentiation program into CD9^+^ TREM2^+^ macrophages, also termed lipid-associated macrophages (LAMs [132];) that are located in crown-like structures in proximity to damaged or dying adipocytes [133, 134]. The TREM2-dependent gene program is required to fully differentiate into LAMs and loss of *Trem2* aggravates obesity in terms of adipocyte hypertrophy, body fat accumulation and weight gain [132], although some non-haematopoietic *Trem2*-dependent effects might contribute to this phenotype [135]. Genes involved in the KEGG pathways lysosome, phagosome, and oxidative phosphorylation further characterize LAMs, while inflammatory genes involved in classical activation were not enriched [132]. These results are in contrast to the notion that adipose macrophages acquire a classical pro-inflammatory phenotype under obese conditions and significantly worsen metabolic function as discussed above. Instead, these findings have brought adipose macrophages into the spotlight for therapeutic strategies by targeting directly or indirectly their metabolic activity [132, 136].

### Atherosclerosis

Foamy macrophages are a distinctive feature of the atherosclerotic plaque and play an important role in their formation, growth, and potential rupture. *Ccl2* as well as *Ccr2* gene deletion halt the progression of dietary-induced atherosclerosis [137, 138]. Subsequent studies showed that monocytes additionally employ CCR5 and CX_3_CR1 receptors to accumulate within lesions and that predominately classical monocytes infiltrate the intima and subintima during atherosclerosis [139]. Recent studies indicate that tissue-resident macrophages in the aortic intima undergo local proliferation. However, in the context of hypercholesterolaemia, these foamy macrophages are replaced by monocyte-derived cells that eventually also adopt a foamy phenotype and gene expression profile similar to those of the previous resident macrophages [140]. Since non-classical monocytes show patrolling behaviour and atherosclerotic lesions affect the arteries, the relevance of these cells during atherogenesis remains under debate. When *Nr4a1*^−/−^  *Apoe*^−/−^ mice—that show reduced numbers of non-classical monocytes—are fed a Western diet, the animals develop aggravated atherosclerosis [141], indicating protective functions of non-classical monocytes. However, *Nr4a1* was also shown to negatively regulate the inflammatory response of classical monocytes [142] and an increased inflammatory profile was evident in the lesions of *Nr4a1*^−/−^  *Apoe*^−/−^ mice [143]. It is therefore possible that once classical monocytes infiltrate into the atherosclerotic plaque they up-regulate *Nr4a1* to limit their activation. The exact contribution of non-classical monocytes requires further investigation, for example mice lacking a specific *Nr4a1* enhancer element that prevents non-classical monocyte generation, but does not limit *Nr4a1* expression under inflammatory conditions [144]. Following the infiltration into lesions, monocyte-derived cells express CD9, genes involved in cholesterol efflux and lipid handling (*Fabp4*, *Trem2*, *Abcg1*), *Lgals3*, *Gpnmb*, *Ctsd*, and *Spp1* [145], which resembles the phenotype of LAMs during obesity. Similar gene expression patterns could be identified in phagocytes isolated from human atherosclerotic vessels [146]. Interestingly, deletion of *Trem2* in myeloid cells reduced plaque deposition and the clinical course of atherosclerosis [147, 148]. These protective effects were attributed to less foamy macrophage generation and reduced survival of plaque macrophages, while systemic inflammation was not affected by *Trem2*-deficiency [147]. Beside lipid-associated cells, macrophages with an inflammatory signature, are evident in mouse and human atherosclerotic plaques [148–150]. Accordingly, deletion of inflammatory genes such as *Myd88*, *Tlr4*, and *Il1b* all diminish plaque formation [151, 152]. As these genetic deletions are not exclusive to monocytes, it remains uncertain which specific subset of macrophages is responsible for mediating the protective effect in atherosclerotic conditions. One could also speculate that circulating lipids may already be impacting the epigenome of blood monocytes, a phenomenon referred to as trained innate immunity [153, 154]. Short-term *in vitro* treatment of monocyte with oxidized low-density lipoprotein (oxLDL) led to enhanced production of TNFα and IL-6, when stimulated 6 days later with TLR2 and TLR4 ligands. Also, the expression of lipid-handling genes (*Cd36*, *Abca1*, *Abcg1*) was increased in these cells, partially due to epigenetic reprogramming [155]. Similarly, hypercholesterolaemia-induced priming of HSC can contribute to atherosclerosis pathology by skewing the haematopoietic output towards the myeloid lineage, thereby aggravating plaque formation in transplanted animals [156].

Further outstanding questions include, how mechanical force in the form of increased sheer stress affects macrophage recruitment and function during atherosclerosis [157]. Monocyte infiltration occurs in atherosclerosis-prone areas of the arterial tree where altered fluid hemodynamics prevail, which makes this area particularly prone to protein deposition as observed in the case of tissue-resident intima macrophage-dependent fibrin accumulation [158]. The build-up of cellular dense plaques and increased sheer pressure from narrowed arteries might also contribute to the activation of mechanosensitive channels for instance *Piezo1*. PIEZO1 has recently been shown to regulate the inflammatory response of innate immune cells during bacterial infection and fibrotic autoinflammation in the lung [159], and its role in atherosclerosis is currently under investigation [160].

Taken together, the molecular mechanisms of atherosclerotic and adipose macrophages during obesity show similarities, although both subsets occupy discrete cellular niches. Defining commonalities and functional differences, in direct comparison to their circulating monocyte counterparts, will enable a clearer understanding of the function these myeloid cells play during metabolic diseases. In addition, a detailed metabolic analysis of different macrophage populations during obesity-related diseases is necessary to study the metabolic reprogramming of macrophages on a subset level. These results should also consider spatial localization and cellular communication between different macrophage populations at various time points during pathogenesis to help unravel the therapeutic value of macrophage reprogramming during metabolic diseases.

## The role of monocytes during autoinflammatory diseases

Monocytes adopt a prominent role as effector cells and contribute to tissue destruction during autoimmune diseases. The depletion of monocytic cells by either non-specific approaches (e.g. clodronate liposomes, constitutive gene deletion) or specific targeted methods (e.g. Cre-dependent diphtheria toxin depletion) reduce clinical symptoms in various autoinflammatory disease models including arthritis [161], diabetic retinopathy [162], glomerulonephritis [163], or systemic lupus erythematosus [164]. These results indicate that the activation of classical monocytes during autoinflammatory disorders significantly contributes to host tissue damage, which has been extensively studied in the mouse model of multiple sclerosis, EAE, and will be used here as an example of the role of monocytes during autoimmunity. An increase in peripheral classical monocytes has been reported during the pre-clinical phase of EAE [112, 165, 166]. Different depletion systems were used to reduce classical monocyte numbers during the priming phase of the disease or at clinical onset to investigate their contribution to pathology. Only the latter treatment approach significantly improved clinical symptoms and reduced tissue damage in the inflamed CNS [112, 166, 167]. Indeed, classical monocytes were the primary infiltrating cells, which were shown to express *Nos2* and *Arg1* in a sequential manner [168]. A subsequent study confirmed the existence of different monocyte-derived cell stages within the CNS and supported the close relationship between *Nos2*- and *Arg1*-expressing cells [56]. Beside these subsets, also *Cxcl10* expression marked a pathogenic population with a pro-inflammatory signature that do not derive from classical monocytes [56]. Short-term monocyte depletion with an antibody against CCR2 reduced the frequency of these cells in the inflamed CNS and correlated with improved clinical symptoms. These data suggest that *Cxcl10*^+^ cells contribute to the pathogenesis in the short-term depletion EAE experiments.

The establishment of EAE is dependent on cytokines such as CSF2 [169] and IL-6 [170], while gene deletion of TNFα, IFNγ, IL-17, IL-22, and IL-12 did not lead to complete EAE protection [171, 172]. Noteworthy though, deletion of TNFα participated in EAE development by the regulation of monocyte survival [167]. It was shown that monocyte-restricted deletion of the *Csf2* receptor (encoded by *Csf2rb*) renders mice resistant to EAE induction, indicating a crucial role for CSF2 signalling in monocyte-derived cells [173]. scRNA-Seq analysis of *Csf2rb*-deficient monocytes in the CNS of mixed bone marrow chimera revealed a *Csf2*-dependent establishment of *Nos2* and *Arg1* expressing cells, while the development of *Cxcl10*^+^ cells was not affected by *Csf2rb*-deficiency [111]. These data emphasizes that *Nos2*^+^ and *Arg1*^+^ myeloid cells are the main drivers of CSF2-dependent CNS tissue destruction, while *Cxcl10*^+^ cells might contribute to clinical manifestation in another way, possibly in the periphery. This is supported by the fact that *Cxcl10*^+^ monocytes could be detected already in the bone marrow independent of immunization [111]. In summary, these data emphasize that GMP-derived cells in the CNS during autoimmune neuroinflammation are CSF2-dependent, thereby excluding MDP-derived cells as CSF2-responsive effector cells within the CNS [111].

The significant involvement of monocytes in the initiation of neuroinflammation could also be observed when CSF2 expression was ectopically targeted to T cells. The expression of CSF2 by T cells was sufficient to generate pathogenic monocytes that cause structural damage within the CNS and cause neuropathological symptoms in an antigen-independent manner [117]. The CNS-infiltrated cells in this model showed a distinct lipid-associated gene signature, which emphasized that an efficient monocytic lipid catabolic activity can contribute to tissue damage in a lipid-rich environment like the CNS. Interestingly, the injection of FLT3L into myelin basic protein-CCL2 transgenic mice causes neuroinflammation with predominantly myeloid infiltrates [174]. If FLT3L/CCL2-educated myeloid cells are transcriptionally comparable and mediate tissue damage in a similar fashion to CSF2-educated cells needs to be shown. It also remains to be shown to what extent CSF2-educated monocytes in the transgenic animal model [117] correspond to CNS-infiltrated monocytes from the classical EAE model.

One question that arises regarding monocytes and neuroinflammtion remains: How do these CNS-infiltrated monocytes contribute to tissue damage? One obvious answer is that monocytes release reactive oxygen species (ROS) through oxidative burst, perform phagocytosis, and secrete cytokines that exhibit neurotoxic activity. However, the deletion of classical ROS-producing enzymes such as myeloperoxidase (*Mpo*) and *Nos2* does not prevent EAE progression in mice and these genes might even play opposing roles during neuroinflammation [175, 176]. However, all CNS-infiltrated monocyte states and subsets show signs of oxidative stress, characterized by the expression of *Cybb*, *Gpx1, Ncf2*, and *Ncf4*, as well as an increased signalling in coagulation and glutathione transferase activity [177]. In addition, *Csf2rb*-dependent cells were enriched in genes involved in inflammasome activation and ROS production, including *Cybb* [111], which might indicate a direct or indirect role for CSF2 in the establishment of oxidative stress response. These pathways possibly lead to the described fibrin deposition and ROS release in EAE lesions that harbour neurotoxic potential. Deletion of *Cybb* in CD11c-expressing cells, which is also expressed in CNS monocyte-derived cells, reduced clinical symptoms during passive EAE [178]. But *Cybb* deletion in *Zbtb46*^Cre^ animals also reduced partially EAE severity [178], indicating a possible role of *Cybb* in cDC rather than monocyte-derived cells.

Another possible mechanism involves the secretion of IL-1β, which is important for the transmigration of monocytes across the blood-brain barrier and the activation of autoreactive CD4^+^ T cells [179, 180]. The important role of IL-1**β** therefore relies on the recruitment of monocytes rather than mediating direct tissue damage.

Taken together, due to their short lifespan and important role in mediating tissue damage, monocytes represent a rational therapeutic target to treat acute relapses of chronic autoimmune diseases.

## Monocytes and antigen presentation

Since the initial observation that bone marrow cells cultured with CSF2 or CSF2 complemented with IL-4 acquire a DC-like phenotype characterized by high CD11c and MHCII expression [181, 182] suggested monocytes participate in antigen presentation. Numerous publications have shown that monocytes can process antigens and accumulate in the inflammatory lesion or in secondary lymphatic organs during inflammatory diseases for a detailed summary in both rodents and humans see [183–185]:. Subsequently, different myeloid depletion systems (such as clodronate liposomes [186, 187] or CD11c-dependent DTR mediated depletion system [188]) or the FLT3L-dependence of DC have been used to discriminate the activities of monocyte-derived cells from the cDC lineage, which led to the conclusion that Ly6C-expressing monocytes efficiently participate in T cell polarization and antigen presentation. It was shown that during EAE monocytes can cross-present antigens [189] and that IFNγ signalling is important to drive the MHCII gene signature in CNS monocyte-derived cells [111]. Thus, CCR2-dependent, pre-clinical deletion of MHCII in monocytes reduced neurological symptoms during EAE, thereby emphasizing a critical role for monocytes in T-cell priming [111]. In a model of DSS-induced colitis, transferred monocytes differentiated into colonic *Cx3cr1*-GFP^Int^ Ly6C^Low^ cells and *Cx3cr1*-GFP^Hi^ Ly6C^Low^ resident macrophages [190]. *Cx3cr1*-GFP^Int^ cells showed expression of *Ccr7*, could be detected in the inflamed colonic lymphatic vasculature, and induced T-cell proliferation *in vitro*.

However, recent advances unravelling myeloid heterogeneity revealed the existence of ‘inflammatory DC2’ and pro-DC3, which both show characteristics of monocytes including expression of Ly6C and CCR2 [23, 191, 192]. Therefore, using Ly6C^+^ CD11b^+^ cells to test the antigen-presenting capacity of monocytes *in vitro* bears the risk of cellular contamination with ‘inflammatory DC2’ and pro-DC3. Purified Ly6C^+^ CD11b^+^ CD115^+^ cells for transfer experiments may contain MDP-derived monocytes or pro-DC3, the latter were shown to harbour antigen presentation capacity and mediate Th17 cell polarizing [23]. Since MDP-derived monocytes showed different migration potential compared to their GMP-derived counterparts [55], it is possible that some of the results mentioned above describe MDP-derived monocytes or pro-DC3 rather than the descendants of classical monocytes. In the future, a careful revaluation of the existing data that specifically target only classical (GMP-derived) monocytes will help elucidate the specific role of monocytes and antigen-presentation.

## Monocytes and the return to healthy homeostasis

Organisms have evolved mechanisms to ensure survival in the face of infection and injury. Regardless the origin of the deleterious stimuli, the fundamental objective of the inflammatory response is to achieve the removal or sequestration of the inciting agent. Inflammation is orchestrated through a series of conserved sequential phases, ultimately leading to tissue remodelling and repair, restoring tissue integrity back to a state of healthy homeostasis. The ‘salutary’ nature of inflammation was recognized over 300 years ago by the father of modern surgery, John Hunter [193]. Wound healing requires the regeneration of damaged cells, while the removal of debris is coordinated by components of the mononuclear phagocyte system. This process is evolutionarily conserved across multicellular organisms and bears a resemblance to developmental biology in certain aspects.

The ongoing discussion in this review regarding the mononuclear phagocyte orchestrators of inflammation, and whether tissue-resident macrophages or blood monocytes are involved in this process, is not a recent quandary. In 1939, Ebert and Florey conducted seminal investigations, tracing the migration of blood monocytes from the circulation to inflammatory sites. Overtime these monocyte-derived cells adopted a morphology reminiscent of macrophages [194] and it was later confirmed that these cells originated in the bone marrow [195, 196]. One exception to this rule occurs in helminth infections, where IL-4 can stimulate the proliferation and the immune activity of resident macrophages independent of recruited monocytes [197]. Nevertheless, in most pathologies, monocyte-derived cells are responsible in the most part in returning tissues back to a healthy state following infection and injury.

The accumulation of cell debris during the inflammatory process ultimately requires its removal in a non-phylogistic fashion [198]. Anti-inflammatory gene expression signatures have been observed in monocyte-derived cells that facilitate tissue regeneration [199–201]. The many faces that infiltrating monocytes may impose throughout the entire inflammatory process, spanning from the genesis to resolution has raised several questions. Do multiple waves of monocyte infiltration occur at distinct phases of the inflammatory response? Or does the same monocyte undertake different functions in a temporal sequence? Trying to answer these questions became even more confusing after the theory of macrophage polarization (M1 and M2) and canonizing the monocyte to an immutable state, as discussed earlier.

Following a skin wound, monocytes and neutrophils enter the site of inflammation almost immediately and represent the first wave of leukocyte immigrants [202]. In injured muscle, heart, intestine, and liver the infiltrating monocyte-derived cells may initially adopt an inflammatory phenotype before helping to restore tissue integrity. Inflammation is a complex process, in turn, monocytes do not simply acquire a binary inflammatory or regulatory phenotype. Instead, the same infiltrating monocyte may respond differently at various stages of the inflammatory process dependent upon the cyto- and chemokines present (Fig. 2). In sterile injury models, such as focussed laser-induced damage to the liver or skeletal muscle injury caused by the tiger snake venom notexin, a myotoxin phospholipase, infiltration of classical monocytes can be observed. Initially, these cells exhibit an inflammatory phenotype and later undergo a transformation into reparative monocytes capable of clearing necrotic debris and orchestrating a return to healthy homeostasis [203, 204]. In the aftermath of skeletal muscle damage, labelled circulating monocytes were shown to migrate to the necrotic site with a pro-inflammatory agenda characterized by high expression of IL-1β and TNFα. Over time, these infiltrating monocytes clear muscle cell debris and transition into regulatory monocyte-derived cells now expressing IL-10 and TGF-β1, with the ability to stimulate myogenesis and contributing to the restoration of muscle integrity [203], which emphasizes that the sweets (cytokines) on offer in the shop changed over time (Fig. 2). A similar dynamic behaviour could be observed in tissue-infiltrated monocytes after myocardial infarction. During the initial state, monocytes exhibit elevated levels of IL-1β, TNFα, and protease activity to facilitate the breakdown of the extracellular matrix. Subsequently, these monocyte-derived cells contribute to tissue remodelling and repair by expressing IL-10, TGF-β1, and VEGF, thereby promoting neoangiogenesis and myofibroblast accumulation [205]. This regulatory behaviour is contingent on the transcription factor NR4A1 [142]. Also following retinal injury, monocyte-derived cells release IL-10 and contribute significantly to the survival and proliferation of retinal progenitor cells for the restoration of homeostasis [206].

The function of monocytes during inflammation and resolution is not only dependent on instructive cyto- and chemokines but also on the concentration of the inflammatory stimulus itself. In a model of skin infection with a high dose of *Staphylococcus aureus*, classical monocyte infiltration is reduced, while neutrophils dominate the inflammatory lesion [118]. In contrast, a low dose of *Staphylococcus aureus* infection led to equal infiltration of classical monocytes and neutrophils. Importantly though, monocytes did not enter the lesion core or contribute to bacteria elimination but rather facilitated neovascularization during the post-infection resolution phase of tissue remodelling [118]. This process was mediated by ghrelin which modulates adipocyte leptin levels, leading to a reduction in vascular overgrowth and promoting efficient wound repair. In the absence of monocytes, a collagenous scar develops and becomes highly vascularized, underscoring the essential role played by monocytes in restoring injured tissue to a state of healthy homeostasis.

The balance between wound healing and fibrosis is a fine tightrope that can be observed in cases of paracetamol-induced liver injury. Infiltrating monocyte-derived cells play a crucial role in hepatic regeneration and the clearance of neutrophils [207, 208]. This was also observed in carbon tetrachloride-induced liver fibrosis, where classical monocytes are recruited to the fibrotic liver and contribute to resolving tissue fibrosis. Their actions involve degrading the extracellular matrix, clearing cellular debris, and expediting scar resolution [200].

These examples show that monocytes have the ability to change from a pro-inflammatory to a regulatory phenotype in response to changes in the microenvironment during distinct phases of the immune response. Circulating monocytes also adopt a regulatory phenotype not only before arriving at the site of inflammation but can be reprogrammed already in the bone marrow. In an oral infection model of *Toxoplasma gondii*, DC in the mesenteric lymph node produces IL-12 that acts in a systemic manner to activate NK cells in the bone marrow, leading to an increase in local IFNγ production. This IFNγ then reprograms monocytes by downregulating the chemokine receptor CX_3_CR1, prompting them to express Sca1^+^, and produce IL-10 and PGE_2_. This immunological circuit ultimately results in regulatory monocytes that prevent lethal pathology by modulating tissue-specific immunity in the gut [209]. In another cellular circuit, monocytes recruited to allergically inflamed skin undergo differentiation to mitigate the allergic response in the presence of basophil-derived IL-4 [210].

These investigations emphasize the plasticity of monocyte-derived cells during the inflammatory response and demonstrate their capacity to undergo a phenotype switch that facilitates the return to a state of healthy homeostasis. At every phase of the immune response, the monocyte-derived cell functions are influenced by its microenvironment, enabling the recovery process. By capitalizing on certain beneficial aspects of the regulatory monocyte-derived cells, we could counter chronic inflammation and facilitate a return to a steady state.

## The role of monocytes in cancer development

At least at the initial stages, solid tumours can be perceived as a newly developing organ [211] that relies on apoptotic cell removal, clearance of cellular debris, angiogenesis, immunosuppression, and remodelling of the extracellular matrix. Tumour macrophages are engaged in all these tasks and the roles adopted by mononuclear phagocytes in solid tumours are therefore analogous to their physiological role in organ development during embryogenesis. Accordingly, tumour macrophages are central contributors to cancer biology.

The solid tumour harbours distinct populations of mononuclear phagocytes that either promote or suppress cancer progression depending on the tumour stage, location, immunogenicity and microenvironment. Both classical monocytes and tissue-resident macrophages have the capacity to transform into tumour-associated macrophages (TAMs) throughout the course of tumour development. The literature commonly refers to both these populations as TAM irrespective of their origin. However, it is possible that each neighbourhood within the tumour dictates a discrete function of the TAM population, as previously described in breast cancer [212]. Monocyte-derived (mo)TAM (for example TREM2^+^ TAM) infiltrate the tumour core, whereas tissue-resident-derived TAMs (such as FOLR2^+^ TAM) are predominately located within the perivascular niche of the tumour stroma [212]. These spatial differences likely result in a different microenvironment and may account for the observed functional differences: while FOLR2^+^ TAMs are strategically positioned to interact with T cells and are accordingly involved in mounting a possible adaptive immune response against the tumour by priming CD8^+^ T cells [212], moTAMs are in direct contact with (apoptotic) tumour cells and experience a hypoxic environment. The clearance of tumour debris and direct signals from tumour cells can contribute to a lipid-associated gene signature and the immunosuppressive state of moTAMs [213]. However, CCR2-dependent moTAMs can also exhibit immune responses by the upregulation of antigen presentation machinery and the release of inflammatory cytokines in T cell-rich pancreatic ductal adenocarcinoma (PDAC) in patients and murine PDAC models, whereas embryonically derived macrophages exhibit a profibrotic signature that contribute to the remodelling of the tumour microenvironment (TME) [214]. In this model, the differentiation of moTAM has recently been shown to follow several stages; initially the infiltrated cells adopt a transient transcriptional profile characterized by *Tnf* and *Ccr2* expression. This population can then further differentiate into two distinct TAM populations, one characterized by *Hif1a* expression and an hypoxic phenotype, while the other shows an immunomodulatory phenotype accompanied by *Trem2* expression. Which differentiation pathway is adopted by the cells is apparently dictated by the microenvironment of the tumour [215]. Indeed, when PDAC monocytes interact with tumour-specific CD4^+^ T cells they can acquire an anti-tumourigenic MHCII^Hi^ phenotype, while in the absence of direct lymphocyte contact, tumour-infiltrated monocytes follow the default differentiation into FOLR2^+^ TAMs, promoting tumour growth [216]. The interplay between myeloid cells and T cells has the potential to promote an ‘anti-tumour’ macrophage response, which may be independent of ontogeny. In contrast, in the absence of such signals, macrophages adopt a default TAM phenotype that supports tumour growth in non-immunogenic cancers.

These examples illustrate the intricate interplay between monocytes with their tumour surroundings and how this influences the function of TAMs. Factors including the rate of cell death, immunogenicity, tissue specificity, localization, immune cell infiltrates, grade of hypoxia, local tumour-derived chemo- and cytokines, macrophage ontogeny, and time all contribute to the heterogeneity of TAMs in different tumours. A detailed understanding of these factors will help to predict TAM functions and may be therapeutically valuable to influence TAM differentiation to favour an immune activation phenotype over an immune suppressive state. Permitting other immune cells like T cells access to the tumour core and thereby allowing them to interact with TAM could further help to modulate and increase the immunsupression state of moTAM.

Maintaining an optimal oxygen concentration is crucial for tissue survival. Tumour hypoxia emerges out of uncontrolled cell proliferation in combination with restricted blood vessel formation, which limits the supply of oxygen. This hypoxic environment prompts responses such as angiogenesis. In the face of hypoxia, recruited monocytes can be activated to participate in angiogenesis, while the hypoxic milieu induces various phenotypic changes. A population of monocytes that express Tie2^+^ has been described as pro-angiogenic [217]. Tie2 appears to be expressed on human intermediate/non-classical human monocytes and is significantly increased in patients with non-small cell lung cancer [218, 219]. However, in a mouse model of angiogenesis, infiltrating classical monocytes undergo transformative changes, acquiring the ability to remodel and generate blood vessels and down-regulate their classical phenotype. Notably, these monocytes appear transient at the angiogenic site and are constantly replenished [220]. In response to low oxygen levels, monocytes up-regulate hypoxia-inducible transcription factors, particularly HIF1α and HIF2α. The myeloid-specific deletion of HIF1α in the murine MMTV-PyMT model reduced tumour growth [221]. Similarly, the absence of HIF2α in TAM showed positive outcomes in hepatocellular and colitis-associated colon carcinomas, associated with a reduced monocyte migration to the tumour [222]. The infiltration of Tie2^+^ VEGFR^+^ CD11b^+^ F4/80^+^ cells in an orthotopic mouse model of glioblastoma occurs also in a HIF1α-dependent manner. These monocyte-derived cells secrete matrix metalloproteinase 9 (MMP9), sufficient to activate the bioavailability of VEGF and turn the angiogenic switch [223]. However, the role of monocytes in facilitating angiogenesis during tumour formation requires further investigation.

## Heterogeneity and roles of monocyte-derived TAMs in tumour progression

A comprehensive profiling of myeloid cells isolated from 15 distinct cancer types indicated that TAM are more heterogenous across each cancer type in comparison to circulating monocytes from the same patients [224], indicating the local TME most likely influences the phenotype of TAM in a complex fashion, similar to the tissue-imprinting of resident macrophages by the organs [97]. Nevertheless, comprehensive scRNA-Seq analysis from different human cancer types was able to classify the possible moTAM populations into two groups: SPP1^+^ macrophages and C1QC^+^ macrophages. Functionally, these two macrophage populations appear to be mutually exclusive [224].

SPP1^+^ macrophages highly upregulate *Vegfa, Marco, Mmp12* genes and express the transcription factors *Cebpb* and *Bhlhe40*. Pathway analysis indicated SPP1^+^ macrophages are associated with tumour angiogenesis, phagocytosis, lipid metabolism, and ECM interactions including the upregulation of metalloproteases [225]. Indeed, they actively interact with cancer-associated fibroblasts or myofibroblast [226]. High expression of *SPP1* correlates with a worse clinical outcome in several cancer types [225] and accordingly SPP1^+^ macrophages are thought to support tumour progression as tumorigenic macrophages.

While C1QC^+^ macrophages significantly increased genes related to phagocytosis compared to SPP1^+^ macrophages [224]. In patients with CRC, RNA velocity analysis of scRNA-Seq data inferred that CD14^+^ monocytes transit to FCN1^+^ monocyte-derived cells, which finally differentiate into C1QC^+^ macrophage subsets [225]. In this study, pathway analysis revealed that C1QC^+^ macrophages show enhanced complement activation and antigen processing/presentation capacity, which emphasizes that they interact with T-cell subsets in patients with CRC. Especially, ligand-receptor pairing analysis of scRNA-Seq showed the enrichment of CXCL10-CXCR3 interaction in C1QC^+^ macrophages, indicating that C1QC^+^ macrophages are involved in T-mediated tumour suppression.

However, in addition to SPP1^+^ and C1QC^+^ TAM populations, tumour-infiltrating monocytes have been identified and described as other cancer-specific moTAM subsets including TREM2^+^ TAM and IL-1β^+^ TAM. TREM*2*^+^ TAMs were initially identified by scRNA-Seq in early lung adenocarcinoma patients [227] and can even be detected at the preclinical stage [228]. These cells further express *CD63*, *SPP1*, and *MARCO* and the presence of this gene signature negatively correlated with patients’ survival, indicating that *TREM2*^+^ TAMs play a similar to SPP1^+^ TAM a pro-tumorigenic and/or immunosuppressive role. Subsequent research showed that the uptake of tumour debris induced the TREM2 gene program in mouse monocyte-derived macrophages (including *Trem2*, *Lpl*, *Cd274*, and *Apoe*), while non-phagocytic cells are immunogenic [213]. Genetic deletion of *Trem2* or therapeutic blockade by anti-TREM2 antibody administration reduced tumour growth in mainly transplantable cancer models [213, 229–231]. In the absence of *Trem2*, TAM in primary lung adenocarcinoma were unable to express IL18bp, which normally intercepts IL-18 and prevents IL-18-dependent activation of NK cells [213]. Accordingly, NK cells show higher anti-tumorigenic activity in *Trem2*-deficient mice. Anti-TREM2 antibody administration during a GL261-induced mouse glioblastoma model also prevents the formation of classical TREM2^+^ TAM and redirects their transcriptomic signature towards pro-inflammatory states [230]. The effectiveness of TREM2 blockade in these models suggests that TREM2^+^ TAM plays a widespread functional role during tumorigenesis, and indeed TREM2^+^ TAM could be detected in a large number of different human carcinoma types, including lung, colon, liver, breast, stomach, and pancreas datasets [231, 232].

Trajectory analysis showed that IL-1β^+^ TAM originates from circulating monocytes and are induced by prostaglandin E_2_ (PGE_2_) secreted by tumour cells in PDAC patients and mice [233]. This loop between IL-1β^+^ TAMs and PDAC shapes the tumour-intrinsic IL-1β response program in hypoxic regions of the pancreatic tumours, resulting in early tumorigenesis. As IL-1β is also highly expressed in PDAC tumour cells [234] and other cancer-associated stromal cells in pancreatic cancer [235, 236], it is possible that PDAC-derived IL-1β can directly induce the proliferation of neighbouring cancer stem cells, resulting in tumour growth. To clearly define whether IL-1β^+^ TAMs are involved in promoting tumours, tumour growth could be assessed in the orthotopic model using IL-1β KO PDCA cell lines or IL-1β depletion in monocyte-derived macrophage models.

 It is currently unclear, how and if TREM2^+^, SPP1^+^, C1QC^+^, and IL-1β^+^ TAM are related or if they describe specific cellular states that depend on pathogenesis and tissue localization. Some literature reports describe an overlapping transcriptomic signature of TREM2^+^ with SPP1^+^ [212], or C1QC^+^ TAM [225] and thereby suggest that some of the markers might describe the same TAM subsets. Recently, *Spp1*-GFP knock-in mice and *Spp1*-CreER mice were generated [237]. These animals are a valuable addition to help reveal the role and fate of SPP1^+^ TAM during tumour progression. In addition, the strong lipid-associated gene pattern observed in TREM2^+^ and SPP1^+^ TAMs suggests that both subsets acquire a phenotype with similar pro-tumorigenic function. An unbiased meta-analysis incorporating all these datasets and establishing a standardized nomenclature will help streamline the categorization and function of the different TAM subsets preventing redundancy and enhancing data set accessibility. This in turn will hopefully aid the development of specific tailored treatments.

## Conclusion

The notion of ‘the monocyte’ has altered considerably over the past 30 years, from a homogeneous population of circulating leukocytes dedicated to macrophage repopulation, to a heterogenous population with a diverse functional repertoire. Recent data suggest that monocyte heterogeneity is already evident at their progenitor level as mouse classical monocytes might emerge via two alternative developmental routes: one differentiation passes through the GMP-cMoP axis, while the other differentiates through MDP. However, it is currently unclear, if both subsets belong to the monocyte lineage or if MDP-derived cells show characteristics of DC. Nevertheless, monocytes are much more than macrophage progenitor cells. Circulating monocytes fulfil a crucial role as a rapid-response unit, orchestrating the initiation and resolution phases of infection, wounds, and disease. These diverse roles share one goal: to restore the physiological balance and a return to tissue equilibrium. Furthermore, monocyte-derived cells can also perform functions that tissue-resident macrophages or DC cannot, although some similarities may occur. Under specific conditions such as spinal cord injury, monocyte-derived cells are able to participate in IL-10-dependent wound healing, while microglia that share the same environment are unable to fulfil these functions [238]. This example demonstrates that the origin-dependent epigenetic imprinting of monocyte permits functions that complement the functions of tissue-resident cells. Yet monocytes are unable to discriminate tumourigenic cells from healthy cells and they can also be easily misled by lymphocytes to mount an overzealous immune response resulting in tissue destruction, as evident in autoinflammatory diseases. Understanding the causes of monocyte infiltration in conjunction with their surrounding microenvironment will aid design future therapeutics that either block or enhance certain monocytic functions. Furthermore, since monocytes are constantly replenished and do not bear a long-lasting genetic immunological memory, the manipulation of circulating monocyte numbers either by their specific depletion, by therapeutics or via specific behaviour such as intermittent fasting, represents an attractive clinical intervention strategy for many acute diseases. Since monocytes can efficiently infiltrate tissues under pathological conditions, they may also be exploited and used therapeutically as ‘Trojan horses’. Accordingly, *ex vivo* manipulation of monocytes might be a suitable approach to equip monocytes with new functions that can beneficially influence the cytokine or cellular milieu of the pathological lesion.

## Data Availability

No new data were generated in support of this review.

## Abbreviations

Arg: arginase
Apoe: apolipoprotein E
BrdU: bromodeoxyuridine
CDP: common DC progenitor
cMoP: common monocyte progenitors
CMP: common myeloid progenitors
CNS: central nervous system
CpG: DNA molecules cytosine triphosphate deoxynucleotide followed by a guanine triphosphate deoxynucleotide
CSF1: colony stimulating factor 1, aka; macrophage colony-stimulating factor (M-CSF)
CSF2: colony stimulating factor 2: aka; granulocyte-macrophage colony-stimulating factor (GM-CSF)
DC: dendritic cell
DTR: diphtheria toxin receptor
EAE: experimental autoimmune encephalomyelitis
EMP: erythro-myeloid progenitors
GFP: green fluorescent protein
GMP: granulocyte-monocyte progenitor
HIF: hypoxia-inducible factor
HSC: haematopoietic stem cells
ICAM: intercellular adhesion molecules
IFN: interferon
IL: interleukin
KEGG: Kyoto encyclopaedia of genes and genomes
LAM: lipid-associated macrophages
LFA: lymphocyte function-associated antigen
LPS: lipopolysaccharides
MBP: myelin basic protein
MDP: monocyte/DC progenitors
MHC: major histocompatibility complex
MMTV-PyMT: mouse mammary tumour virus-polyoma Virus middle T antigen
MMP: metalloproteinase
MPO: myeloperoxidase
MPP: multi-potent progenitors
MPS: mononuclear phagocyte system
NOS: nitric oxide synthase
NSCLC: non-small cell lung cancer
oxLDL: oxidized low-density lipoprotein
PDAC: pancreatic ductal adenocarcinoma
pDC: plasmacytoid dendritic cells
PGE_2_: prostaglandin E2
ROS: reactive oxygen species
scRNA-seq: single cell RNA sequencing
SPF: specific pathogen-free
TAM: tumour-associated macrophages
Th: T helper
TLR: toll like receptor
TME: tumour microenvironment
TNF: tumour necrosis factor
VEGF: vascular endothelial growth factor
WT: wild type
